# Allosteric Modulators of the CB_1_ Cannabinoid Receptor: A Structural Update Review

**DOI:** 10.1089/can.2015.0005

**Published:** 2016-01-01

**Authors:** Paula Morales, Pilar Goya, Nadine Jagerovic, Laura Hernandez-Folgado

**Affiliations:** Instituto de Química Médica, Consejo Superior de Investigaciones Científicas, Madrid, Spain.

**Keywords:** CB_1_R, synthetic cannabinoids

## Abstract

In 2005, the first evidence of an allosteric binding site at the CB_1_R was provided by the identification of three indoles of the company Organon that were allosteric enhancers of agonist binding affinity and, functionally, allosteric inhibitors of agonist activity. Since then, structure–activity relationships of indoles as CB_1_R modulators have been reported. Targeting the allosteric site on CB_1_R, new families structurally based on urea and on 3-phenyltropane analogs of cocaine have been discovered as CB_1_R-negative allosteric modulators (NAMs), respectively, by Prosidion and by the Research Triangle Park. Endogenous allosteric ligands of different nature have been identified more recently. Thus, the therapeutic neuroprotection application of lipoxin A4, an arachidonic acid derivative, as an allosteric enhancer of CB_1_R activity has been confirmed *in vivo*. It was also the case of the steroid hormone, pregnenolone, whose negative allosteric effects on *Δ*^9^-tetrahydrocannabinol (*Δ*^9^-THC) were reproduced *in vivo* in a behavioral tetrad model and in food intake and memory impairment assays. Curiously, the peroxisome proliferator-activated receptor-γ agonist fenofibrate or polypeptides such as pepcan-12 have been shown to act on the endocannabinoid system through CB_1_R allosteric modulation. The mechanistic bases of the effects of the phytocannabinoid cannabidiol (CBD) are still not fully explained. However, there is evidence that CBD behaves as an NAM of *Δ*^9^-THC- and 2-AG. Allosteric modulation at CB_1_R offers new opportunities for therapeutic applications. Therefore, further understanding of the chemical features required for allosteric modulation as well as their orthosteric probe dependence may broaden novel approaches for fine-tuning the signaling pathways of the CB_1_R.

## Introduction

One traditional way of designing new drugs is the so-called one target–one disease approach. Indeed, this is the case of many commercially available drugs, which interact with enzymes, receptors, or ionic channels among others. One of the most important targets are the G-protein-coupled receptors (GPCRs) for which agonists and antagonist/inverse agonists have been developed.

These traditional ligands bind to the same site as the endogenous ligand, the so-called orthosteric site. However, recently, research has shifted to compounds that can interact with a different region of the receptor, termed the allosteric site, since this approach may result in pharmacological advantages such as higher specificity and thus reduced side effects. The best known mechanisms of action of these allosteric ligands are activation or inhibition of the receptor signaling, referred to as positive allosteric modulators (PAMs) and negative allosteric modulators (NAMs). There are also neutral or silent ligands, silent allosteric modulators, which do bind to the allosteric site without affecting the response of the endogenous agonist. In addition, bitopic ligands that bind to both the orthosteric and allosteric sites have been described. In the last years, there has been considerable research in allosteric modulation of GPCRs both in academia and industry,^1,2^ and structural knowledge on how these interactions take place has advanced on the basis that some cocrystals of a ligand bound to an allosteric site have been obtained.^3^

In addition to two allosteric modulators marketed for HIV and hyperparathyroidism, recent potential therapeutic applications of allosteric modulators of GPCRs include central nervous system (CNS) disorders such as neurodegenerative diseases or schizophrenia^4^ and also pain for which PAMs of the μ-opioid receptor have been proposed.^5^

A particular kind of GPCRs are the cannabinoid CB_1_ and CB_2_ receptors (CB_1_R and CB_2_R), for which thousands of traditional ligands, belonging to many different chemical structures, have been synthesized and evaluated. However, this has had only limited success in bringing them to the clinic. In fact, only three natural derived medicines (Marinol™, Nabilone™, and Sativex™) are available largely due to their unwanted side effects.

One possibility to overcome the limitations of these traditional cannabinoids interacting with the orthosteric sites would be to use allosteric cannabinoid ligands. Despite the considerable research undertaken on traditional cannabinoid agonists and antagonists, there have been comparatively few reports on allosteric cannabinoid ligands. In this review, we will give an overview of the chemical structures that have been tested and proven to be allosteric cannabinoid ligands, but will not deeply deal with the pharmacological aspects of cannabinoid allosterism, which has been covered elsewhere.^6–9^ According to structural features, CB_1_R allosteric modulators reported to date can be classified as follows: indole derivatives; urea derivatives; and structures acting as endogenous ligands and miscellaneous structures.

## Indole Derivatives

**FIG. 1.**
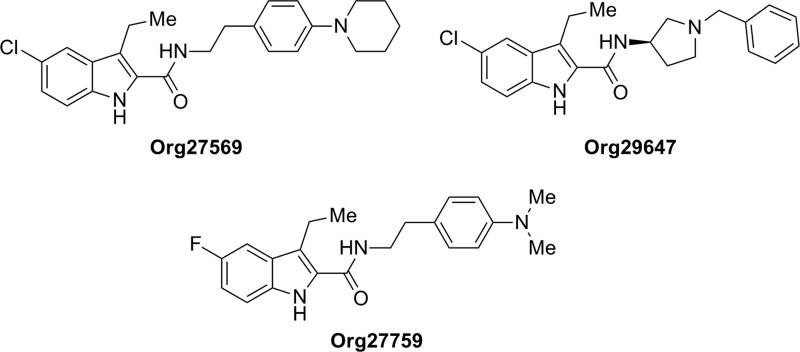
Structure of indoles Org27569 (pharmacological profile in Table 1), Org29647, and Org27759.

The first evidence for an allosteric binding site on CB_1_R was revealed in 2005 by pharmacological studies realized on three indoles from the company Organon Research, Org27569, Org29647, and Org27759 (Fig. 1).^10^ Among them, Org27569 has been more widely studied and is considered as a prototypical CB_1_R allosteric modulator. Org27569 behaves as a positive allosteric enhancer of agonist binding, whereas in terms of functionality, Org27569 is considered a NAM. Lately, the effect of Org27569 as a NAM has been confirmed in a neuronal model of endocannabinoid synaptic transmission.^11^ However, when tested *in vivo*, Org27569 lacked efficacy in modulating the action of orthosteric cannabinoids.^12^ Thus, controversial pharmacological data led to a very recent study in which the allosteric effects of Org27569 at CB_1_R have been revealed to be pathway specific and time dependent, suggesting a more complex process than initially proposed.^13^

The Org27569-binding site has been determined by Shore et al.^14^ using a combination of molecular modeling, mutation, and functional assays. This site was identified in the CB_1_R transmembrane helix (TMH)3–6–7 region. Org27569 acts by modulating receptor activity through conformational changes in the receptor. The authors described how Org27569 sterically blocked movements of the extracellular loops and TMH6 that are involved in receptor activation. Recently, site-directed fluorescence labeling studies reported by Fay et al.^15^ showed that effectively TMH6 movements are associated with G-protein activation, whereas its attached helix eight could be involved in the binding of arrestin triggering the biased signaling pathway.

**FIG. 2.**
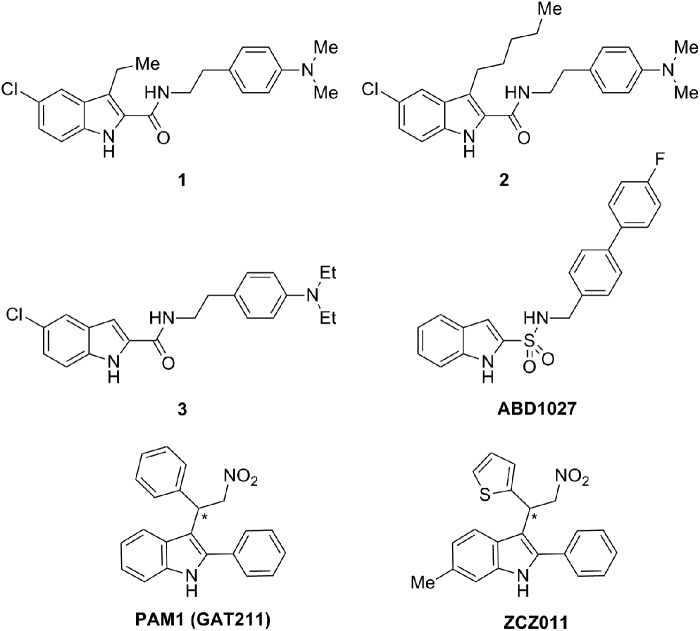
Indole derivatives as CB_1_R allosteric modulators: **1**, **2**, **3**, ABD1027, PAM1 (GAT211), and ZCZ011 (pharmacological profile in Table 1). *Race MIC mixture.

Following discovery of Org27569, structure–activity relationship (SAR) studies were reported by various research groups confirming that indole-2-carboxamide was a good scaffold for developing allosteric modulators for CB_1_R. However, it was only in 2012 that Piscitelli et al.^16^ reported that the first indole-2-carboxamides structurally correlated with Org27569. The very close derivative of Org27759, the indole **1** (Fig. 2), was found to possess the highest positive stimulation of the series on the orthosteric agonist CP-55,940 binding at CB_1_R.

A large number of Org27569 analogs have been identified in Kendall and Lu's laboratories.^17–19^ Among them, it is worthy to mention compound **2**, the 3-pentyl-1*H*-indole-2-carboxamide derivative (Fig. 2), as an allosteric enhancer of [^3^H]-CP-55,940 binding with a markedly high binding cooperativity factor and potent antagonism of agonist-induced GTPγS binding.^18^ Interestingly, the hexyl and the propyl derivatives of **2** induced β-arrestin 1-mediated pathway-biased signaling.^19^

Very recently, a novel series of 1*H*-indole 2-carboxamides has been reported by Nguyen et al.^20^ The most potent compound **3** (Fig. 2) is reported as a NAM of CP-55,940 at CB_1_R according to calcium mobilization assays.

After a first generation of Org27569 derivatives, new series of 2-phenyl-1*H*-indoles have been identified as PAMs at CB_1_R. Compound PAM1, also named as GAT211, displayed in Figure 2, exemplifies the series of 3-(2-nitro-1-arylethyl)-1*H*-indoles claimed by Thakur et al.^21^ as PAMs for CB_1_R in cAMP functional assays. Due to the presence of a chiral center, GAT211 is a racemic mixture of two optical isomers (R and S) that were isolated using supercritical fluid chromatography. Difference in pharmacology potencies of both enantiomers could be appreciated.

Ignatowska-Jankowska et al.^22^ reported the pharmacological properties of 6-methyl-3-[2-nitro-1-(thiophen-2-yl)ethyl]-2-phenyl-1*H*-indole (ZCZ011; Fig. 2). ZCZ011 increased [^3^H]-CP-55,940 binding and potentiated anandamide (AEA)-stimulated signaling in [^35^S]-GTPγS binding, β-arrestin recruitment, and extracellular signal-regulated kinase (ERK) phosphorylation assays. A very interesting finding is that PAM effects of ZCZ011 could be confirmed by *in vivo* assays. The indole ZCZ011 exhibited antinociceptive effects in neuropathic and inflammatory pain models with no associated cannabimimetic effects.

Greig et al.^23^ from the University of Aberdeen applied the bioisosteric replacement of carboxamide by sulfonamide leading to numerous *N*-arylalkyl-1*H*-indole-2-sulfonic amides claimed in a patent. Inventors reported that ABD1027 (Fig. 2) did not affect [^3^H]-CP-55,940 binding, although it inhibited agonist signaling in functional β-arrestin recruitment assays.

## Urea Derivatives

**FIG. 3.**
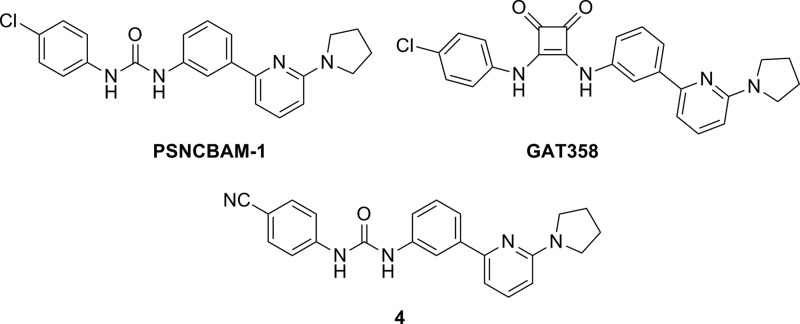
PSNCBAM-1 and its analogs as CB_1_R allosteric modulators: GAT358 and **4** (pharmacological profile in Table 1).

In 2007, the company Prosidion identified a novel class of CB_1_R allosteric compounds through high-throughput screening of a small library.^24^ The lead optimized urea of this series, PSNCBAM-1 (Fig. 3), increased CB_1_R agonist binding and behaved as an allosteric antagonist in [^35^S]-GTPγS binding and cAMP assays. In this study, acute food intake experiments provided the first *in vivo* data showing the efficacy of CB_1_R allosteric antagonism similar to the antiobesity effects of the well-known CB_1_R antagonist, SR141716.

In 2015, 8 years after the discovery of PSNCBAM-1, the first SAR studies on PSNCBAM-1 were published by German et al.^25^ The resulting analogs showed similar pharmacological profiles to the parent NAM in binding and calcium mobilization assays. Structural modifications have focused on the pyridine and the 4-chlorophenyl groups. Substituted amine with small size alkyl chains showed to be preferred for pyridine substitution. Substitution in position 4 of the phenyl ring with an electrowithdrawing group was revealed to be important for activity (compound **4**; Fig. 3).

In the same year, Thakur et al.^26^ claimed novel CB_1_R allosteric modulators based on PSNCBAM-1 structure. SAR studies involving bioisosterism of urea were extensively examined with the synthesis and evaluation of carbamates, thioureas, 1,3,4-oxadiazol-2-amines, and 3,4-diaminocyclobut-3-ene-1,2-diones, leading to functionally selective NAMs. The 3,4-diaminocyclobut-3-ene-1,2-dione derivative, GAT358, was selected for *in vivo* behavioral tests that suggested minimal CB_1_R inverse agonist-related side effects.

## Endogenous CB_1_R Allosteric Modulators

In addition to synthetic allosteric modulators categorized herein, endogenous molecules of diverse chemical nature have been identified as allosteric modulators of CB_1_Rs.

**FIG. 4.**
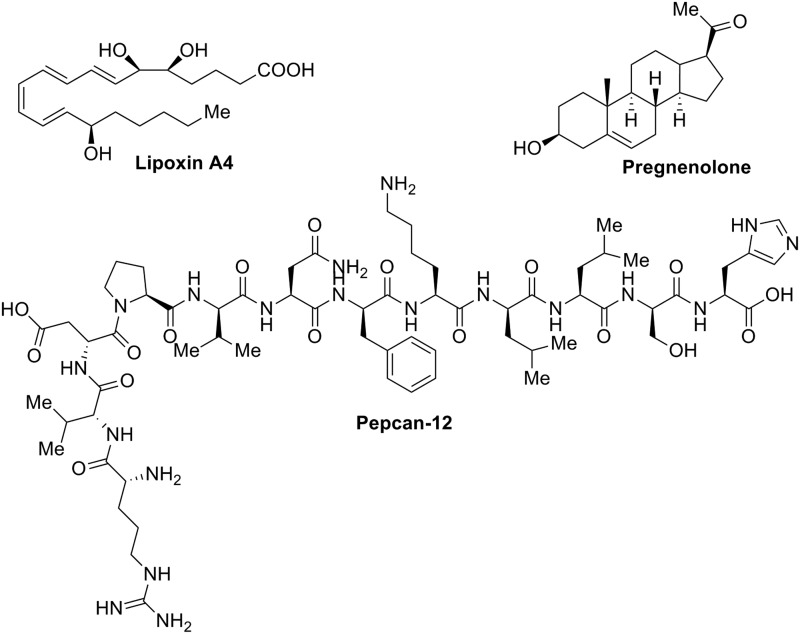
Endogenous CB_1_R allosteric modulators: lipoxin A4, pregnenolone, and pepcan-12 (pharmacological profile in Table 1).

One of these molecules is lipoxin A4 (Fig. 4), an oxygenated derivative of arachidonic acid involved in immune system regulation and known as a potent endogenous anti-inflammatory mediator. However, the specific effects of lipoxin A4 in the CNS were reported to be mediated by unknown mechanisms. In 2012, lipoxin A4 was proposed as an allosteric modulator of CB_1_R by Pamplona et al.^27^ This lipid acted as a CB_1_R PAM, enhancing receptor binding of AEA and [^3^H]-CP-55,940 and not only potentiating selectively AEA- versus 2-AG in HEK293-CB_1_R cells but also in the behavioral tetrad tests. The authors confirmed the therapeutic application for neuroprotection of lipoxin A4 as an allosteric enhancer of CB_1_R activity in an *in vivo* model of β-amyloid-induced spatial memory impairment. Recently, Staiker et al.^11^ reported that lipoxin A4 surprisingly exhibited a CB_1_R NAM profile, and no PAM as reported so far, in a neuronal model of 2-AG-mediated depolarization-induced suppression of excitation (DSE). Thus, this effect could be attributable to a potential probe dependence of lipoxin A4. Adding to the complexity, recent studies realized by Khajehali et al.^13^ on lipoxin A4 could not corroborate the PAM modulatory effects on either AEA- or CP-55,940-mediated cAMP inhibition in CHO-CB_1_R cells.

Another putative endogenous allosteric modulator at the CB_1_R is the steroid hormone pregnenolone (Fig. 4), a hydrophobic precursor for all C_18_, C_19_, and C_21_ steroids directly synthesized from cholesterol. The therapeutic known targets for pregnenolone are GABA and NMDA receptors.^28^ However, in 2014, Vallée et al.^29^ identified pregnenolone as an allosteric inhibitor at CB_1_Rs decreasing *Δ*^9^-tetrahydrocannabinol (*Δ*^9^-THC)-induced ERK1/2 phosphorylation in CHO-CB_1_R cells. Furthermore, these NAM effects on *Δ*^9^-THC were reproduced *in vivo* in a behavioral tetrad model, in food intake and memory impairment assays. In contrast, in the neuronal model reported by Staiker et al.,^11^ pregnenolone failed to modulate 2-AG of synaptic transmission. Likewise, Khajehali et al.^13^ reported lack of modulatory effect of pregnenolone on either *Δ*^9^-THC- or WIN55,212–2-induced activation of ERK1/2 phosphorylation. These divergent data suggest probe- or pathway-dependent allosteric effects.

Until recently, synthetic or endogenous compounds acting on the endocannabinoid system (ECS) were of lipid nature. In 2012, Bauer et al.^30^ described a 12-amino acid sequence, structurally corresponding to an *N*-terminal extended form of hemopressin, as a potent CB_1_R NAM of orthosteric agonist-induced cAMP accumulation, [^35^S]-GTPγS binding, and receptor internalization. This polypeptide, named pepcan-12, has been recently localized in discrete cells of the CNS and adrenal gland.^31^ It is worth mentioning that this endocannabinoid peptide was previously reported to interact with CB_1_R as an agonist.^32^ Furthermore, the NAM profile of pepcan-12 has been confirmed in the DSE study of 2-AG synaptic transmission performed by Staiker et al.^11^

## Miscellaneous CB_1_R Allosteric Modulators

The interest gained in this field led to the identification of an assorted range of other small molecules that also display CB_1_R allosteric modulation.

**FIG. 5.**
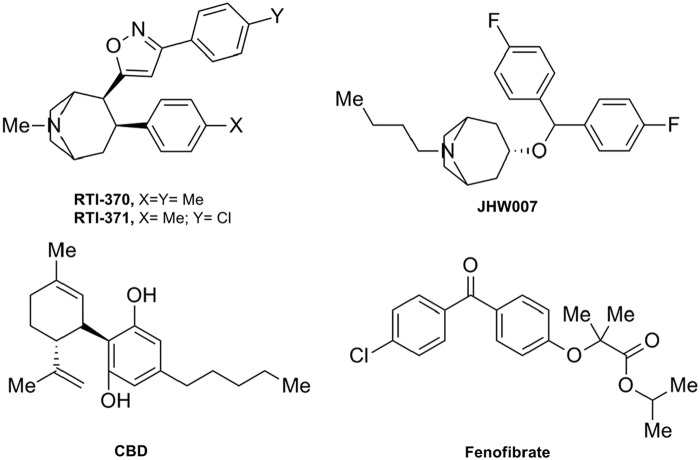
Miscellaneous CB_1_R allosteric modulators: RTI370, RTI371, JHW007, cannabidiol (CBD), and fenofibrate (pharmacological profile in Table 1).

In 2009, researchers from the Research Triangle Park screened several 3-phenyltropane analogs of cocaine for their activity at the CB_1_R.^33^ These molecules had been previously characterized as dopamine transporter (DAT) inhibitors.^34^ Among this series, the tropanes, RTI-371 and RTI-370, and the benztropine JHW007 (Fig. 5) stand out as PAMs of CP-55,940-induced CB_1_R activity in a cell-based calcium mobilization assay. RTI-371 was found to be of special interest because it is a potent and selective DAT inhibitor that lacks cocaine-like behavioral effects and abuse liability. According to the authors, these atypical cocaine-related effects could be due to positive allosteric modulation of the CB_1_R.^33^ This study highlights the potential of the 3-phenyltropane scaffold for the development of novel CB_1_R PAMs.

The nonpsychoactive phytocannabinoid cannabidiol (CBD; Fig. 5) has shown diverse promising therapeutic applications through multiple biological targets. CBD is known to interact with many nonendocannabinoid signaling systems such as the opioid receptors, the transient channel receptor, transient receptor potential channel 1, or the nuclear receptor, peroxisome proliferator-activated receptor (PPAR)-γ, among others. CBD does not bind to the orthosteric CB_1_R binding site; however, it has been shown to display antagonism of CB_1_R agonists *in vitro*.^35–37^ More recently, Laprairie et al.^38^ provided evidence that CBD behaved as an NAM of *Δ*^9^-THC- and 2-AG-dependent CB_1_R internalization, β-arrestin recruitment, and phospholipase C β3- and ERK1/2-phosphorylation. These results may explain some of the *in vivo* effects of this promising nonpsychoactive compound providing novel insights in the intriguing pharmacology of CBD.

The close relationship between the ECS and the PPARs led to explore the activity of the PPAR-α agonist, fenofibrate^39^ (Fig. 5), in the ECS. This fibrate is an amphipathic molecule that acts as a prodrug formed by fenofibric acid linked to an isopropyl ester. Priestley et al.^40^ reported the pharmacological profile of fenofibrate at CBRs. [^35^S]-GTPγS binding experiments revealed partial agonism of fenofibrate at CB_1_R and full agonism at CB_2_R. Moreover, at higher concentrations, this PPAR-α ligand was also able to significantly decrease [^35^S]-GTPγS binding of CP-55,940 acting as an NAM of CB_1_R. Thus, the authors suggested two possible interpretations. Fenofibrate could be considered a bitopic ligand at CB_1_R since it binds both orthosteric and allosteric sites or fenofibrate could be an allosteric CB_1_R modulator that could be able to produce by itself a functional response by interacting with a nonorthosteric site at CB_1_R.

## Conclusions and Perspectives

The psychoactive side effects generated by activation of CB_1_R in the brain have limited the use of orthosteric CB_1_R ligands as drugs. However, this receptor plays an important role in diverse processes such as pain, cognition, and metabolism. Alternative targets within the ECS have been proposed, including CB_2_R, the fatty acid amide hydrolase, or the monoacylglycerol lipase. Allosteric modulation at CB_1_R offers new opportunities for therapeutic applications. Even though the first CB_1_R allosteric ligand, Org27569, was identified in 2005, significant interest by the research community only started few years ago. Then, there have been rapidly increasing research efforts directed into CB_1_R allosterism during these last 3 years. This review reflects the structural diversity and nature of the CB_1_R allosteric modulators identified so far. In particular, there has been an increasing identification of endogenous allosteric modulators that offer new insights in the intriguing pharmacology of CB_1_R. In Table 1, a summary of the pharmacological profile of diverse CB_1_R allosteric modulators is displayed. It is worth mentioning that in several of the studies discussed above, there is no correlation between the different cell-based pharmacological data. Moreover, the *in vitro* data do not always translate to *in vivo* effects. These divergent functional data may account for signaling-specific allosteric modulation as well as orthosteric probe dependence. Subsequently, there is a clear need for developing more allosteric pharmacological tools for understanding this complex pharmacology.

**Table 1.** **Summary of CB_1_R Allosteric Modulators and Their Cannabinoid Pharmacological Profile**

			CB_1_R allosterism	
Compound	CB_1_R/CB_2_R orthosteric modulation	CB_1_R allosteric modulation	Functional outcome (orthosteric ligand)	References
Org27569	CB_1_R inverse agonist^6^	NAM	[^35^S]-GTPγS binding assay^a^ (CP-55,940; AEA), cAMP assay, β-arrestin recruitment^a^ (CP-55,940; WIN55,212, AEA)	Price *et al.* 2005^10^
		PAM	ERK1/2 phosphorylation^a^ (CP-55,940)	Baillie *et al.* 2013^6^
		NAM	ERK1/2 phosphorylation^a^ (2-AG)	Khajehali *et al.* 2015^13^
		NAM	DSE^c^ (2-AG)	Straiker *et al.* 2015^11^
2	—	NAM	[^35^S]-GTPγS binding assay^b^ (CP-55,940)	Mahmoud *et al.* 2013^18^
3	—	NAM	Ca^2+^ mobilization assay^a^ (CP-55,940)	Nguyen *et al.* 2015^20^
PAM1 (GAT211)	—	PAM	cAMP assay (CP-55,940)	Thakur *et al.* 2013^21^
ZCZ011	CB_1_R agonist^22^	PAM	[^35^S]-GTPγS binding assay^d^ (CP-55,940; AEA)	Ignatowska-Jankowska *et al.* 2015^22^
		NE	cAMP assay^a^ (CP-55,940; AEA)	
		PAM	ERK1/2 phosphorylation^a^ (CP-55,940; AEA)	
		PAM	β-arrestin recruitment^b^ (AEA)	
ABD1027	—	NAM	β-arrestin recruitment^b^ (CP-55,940)	Greig *et al.* 2012^23^
PSNCBAM-1	CB_1_R partial inverse agonist^24^	NAM	[^35^S]-GTPγS binding assay^b^ (CP-55,940; AEA), cAMP assay^b^ (CP-55,940; AEA)	Horswill *et al.* 2007^24^
		NAM	[^35^S]-GTPγS binding assay^e^ (CP-55,940; WIN55,212)	Wang *et al.* 2011^41^
		NAM	β-arrestin recruitment^a^ (CP-55,940; WIN55,212)	Baillie *et al.* 2013^6^
		NAM	DSE^c^ (2-AG)	Straiker *et al.* 2015^11^
4	—	NAM	Ca^2+^ mobilization assay^f^ (CP-55,940)	German *et al.* 2014^25^
GAT358	—	NAM	cAMP assay (CP-55,940), β-arrestin recruitment^b^ (CP-55,940)	Thakur *et al.* 2015^26^
Lipoxin A4	NE^27^	PAM	cAMP assay^b^ (AEA)	Pamplona *et al.* 2012^27^
		NAM	DSE^c^ (2-AG)	Straiker *et al.* 2015^11^
		NE	cAMP assay^a^ (AEA; CP-55,940)	Khajehali *et al.* 2015^13^
Pregnenolone	—	NAM	ERK1/2 phosphorylation^a^ (*Δ*^9^-THC)	Vallée *et al.* 2014^29^
		NE	DSE^c^ (2-AG)	Straiker *et al.* 2015^11^
		NE	ERK1/2 phosphorylation^a^ (*Δ*^9^-THC, WIN55,212)	Khajehali *et al.* 2015^13^
Pepcan 12	CB_1_R agonist^32^	NAM	cAMP assay, [^35^S]-GTPγS binding, CB_1_R internalization^a^ (2-AG, WIN55,212, HU210)	Bauer *et al.* 2012^30^
		NAM	DSE^c^ (2-AG)	Straiker *et al.* 2015^11^
RTI-371	NE^33^	PAM	Ca^2+^ mobilization assay^a^ (CP-55,940)	Navarro *et al.* 2009^33^
CBD	Weak CB_1_R/CB_2_R antagonist^36,37^	NAM	PLCβ3- and ERK1/2 phosphorylation, β-arrestin recruitment^b,g^ (*Δ*^9^-THC, 2-AG)	Laprairie *et al.* 2015^38^
Fenofibrate	CB_1_R partial agonist/CB_2_R agonist^40^	NAM	[^35^S]-GTPγS binding^a^ (CP-55,940)	Priestley *et al.* 2015^40^

^a^ CHO-CB_1_R cells.

^b^ HEK293-CB_1_R.

^c^ Hippocampal neurons.

^d^ Mouse brain membranes.

^e^ Cerebellar membranes.

^f^ RD-HGA16-CB_1_R cells.

^g^ STH*dh*^Q7/Q7^-CB_1_R.

*Δ*^9^-THC, *Δ*^9^-tetrahydrocannabinol; AEA, anandamide; CBD, cannabidiol; DSE, depolarization-induced suppression of excitation; ERK, extracellular signal-regulated kinase; NAM, negative allosteric modulator; NE, no effect; PAM, positive allosteric modulator; PLC, phospholipase C.

The characterization of small molecules as allosteric modulators of CB_1_Rs provides the basis for future design and synthesis of optimized allosteric modulators. Efforts need to be made to expand these scaffolds and obtain structural information about the ligand–allosteric site interactions. Further understanding of the chemical features required for allosteric modulation as well as their orthosteric probe dependence may broaden novel approaches for fine-tuning the signaling pathways of the CB_1_R.

## Abbreviations Used

2-AG: 2-arachidonylglycerol
AEA: anandamide
CB_1_R: cannabinoid receptor 1
CB_2_R: cannabinoid receptor 2
CBD: cannabidiol
CNS: central nervous system
DAT: dopamine transporter
DSE: depolarization-induced suppression of excitation
ECS: endocannabinoid system
ERK: extracellular signal-regulated kinase
GPCR: G-protein-coupled receptor
NAM: negative allosteric modulator
PAM: positive allosteric modulator
PLC: phospholipase C
PPAR: peroxisome proliferator-activated receptor
SAR: structure–activity relationship
TMH: transmembrane helix
*Δ*^9^-THC: *Δ*^9^-tetrahydrocannabinol
